# Pharmacist-Led Follow-Up After Off-Label Cholestyramine Use for Suspected Bile Acid Diarrhea in a Patient With a Prior Cholecystectomy: A Case Report

**DOI:** 10.7759/cureus.115557

**Published:** 2026-08-31

**Authors:** Yasunari Kinoshita

**Affiliations:** 1 Community Pharmacy, Welcia Pharmacy Co. Ltd., Tokyo, JPN

**Keywords:** bile acid diarrhea, cholestyramine, off-label drug use, pharmacist follow-up, tracing report

## Abstract

Bile acid diarrhea (BAD) is a condition in which excessive bile acids reach the colon, promoting water and electrolyte secretion and potentially causing diarrhea, and is known as one possible mechanism of post-cholecystectomy diarrhea. This case involved a 59-year-old man who had undergone cholecystectomy approximately 18 years earlier and had persistent loose stools and increased stool frequency. BAD was suspected by the treating physician, although it was not definitively confirmed by specific diagnostic testing. The patient was prescribed Questran powder 44.4% at 18 g/day. However, in Japan, the approved indications for Questran powder 44.4% include hypercholesterolemia, and its use for suspected BAD is off-label. The pharmacist was not involved in the diagnosis or treatment selection but reviewed domestic and overseas drug information as support for appropriate use after off-label prescribing. Based on the United Kingdom Summary of Product Characteristics, the pharmacist explained the administration method, the need to separate from concomitant medications, and the need for early follow-up. Before treatment, the patient-reported Bristol Stool Form Scale (BSFS) score was 5-6, and stool frequency was five to six times per day. On day 5, after treatment initiation, pharmacist follow-up showed improvement to a BSFS score of 4-5 and stool frequency of approximately two to three times per day. However, the patient reported difficulty taking the suspension because of its texture. Although the prescription instructed Questran powder 44.4% 18 g/day in two divided doses, the patient maintained the prescribed total daily amount of 18 g/day but took it in three divided administrations. The pharmacist provided the treating physician with information on symptom changes, administration difficulties, and discrepancies between the prescribed regimen and actual medication use via a tracing report. This single uncontrolled observation cannot establish the efficacy of cholestyramine or confirm BAD. Rather, this case illustrates the potential value of pharmacist-led follow-up after off-label prescribing, including early monitoring, identification of medication-use problems, and communication with the treating physician.

## Introduction

Post-cholecystectomy diarrhea is a clinical syndrome that may occur after cholecystectomy, and bile acid diarrhea (BAD) is one possible underlying mechanism. BAD is a condition in which excessive bile acids flow into the colon, promoting water and electrolyte secretion and causing chronic diarrhea. After cholecystectomy, the storage function of the gallbladder is lost, and BAD may occur through changes in bile acid metabolism and intestinal motility. BAD is also recognized as one of the differential diagnoses of chronic diarrhea [1-3].

Cholestyramine, a bile acid sequestrant, is widely used to treat BAD [1]. In contrast, in Japan, the approved indications for Questran powder 44.4% include hypercholesterolemia, and its use for BAD is off-label [4]. By comparison, the United Kingdom Summary of Product Characteristics (SmPC) states that cholestyramine is used for diarrhea associated with ileal resection, Crohn’s disease, vagotomy, and diabetic vagal neuropathy, and that alternative therapy should be initiated if a therapeutic response is not observed within three days in diarrhea caused by bile acid malabsorption [5].

In off-label prescribing, it is important to continuously evaluate not only the pharmacological rationale of the medication but also the appropriate administration method, interactions with concomitant medications, early therapeutic response, adverse events, and actual medication use. Therefore, for patients with suspected BAD prescribed cholestyramine off-label, specifically describing how pharmacists supplement drug information, evaluate medication use and symptom changes, identify medication-use problems, and provide information to physicians through a tracing report has practical significance in community pharmacy practice.

In this case, cholestyramine was prescribed off-label to a patient with a history of cholecystectomy in whom the treating physician suspected BAD. The pharmacist was not involved in the diagnosis or treatment selection, but, from the standpoint of support for appropriate use, reviewed domestic and overseas drug information and conducted early medication follow-up using the Bristol Stool Form Scale (BSFS) and stool frequency. We report this case as a practical example in which the pharmacist identified medication-use problems and provided the resulting information to the physician through a tracing report after off-label prescribing for suspected BAD.

## Case presentation

The patient was a 59-year-old man with a history of cholecystectomy approximately 18 years earlier. In early February, while on a business trip to China, he developed herpes zoster and noticed a mild tendency toward loose stools after taking antiviral medications and other drugs. Subsequently, at a follow-up visit after returning to Japan, gastrointestinal medications were prescribed for his gastrointestinal symptoms.

The symptoms temporarily improved; however, in late February, after eating high-fat foods including oysters and grilled meat, he developed watery stools. At that time, he had no nausea, vomiting, or fever. At a medical institution, norovirus infection was initially suspected, and probiotics and other medications were prescribed. The following day, because bacterial gastroenteritis could not be ruled out, an antibacterial agent was added. However, no secondary infections were observed among the patient’s family members or in his workplace. Beyond the history of cholecystectomy and recent herpes zoster, no additional comorbidities relevant to persistent diarrhea were documented in the information available to the pharmacist. Information on other alarm features was not available to the pharmacist beyond the absence of nausea, vomiting, and fever.

Because the tendency toward loose stools persisted, he visited his primary care physician in late March. As the cause was unclear, he was followed up with a lactic acid bacterial preparation. He had also been informed that the clinical course might differ from typical infectious gastroenteritis because the watery stools that occurred in February were not accompanied by associated symptoms such as nausea, vomiting, or fever.

Thereafter, based on his history of cholecystectomy and the persistence of loose stools and diarrhea after ingestion of high-fat foods, the treating physician suspected BAD rather than definitively diagnosing it. In the information available to the pharmacist from routine community pharmacy practice, no results of relevant laboratory or diagnostic investigations for persistent diarrhea were available. Specifically, no results of stool culture, blood tests, abdominal imaging, colonoscopy, 75-selenium homocholic acid taurine (SeHCAT) testing, serum 7α-hydroxy-4-cholesten-3-one, fibroblast growth factor 19, or fecal bile acid measurement were available to the pharmacist. Therefore, BAD was not definitively confirmed and remained a clinical suspicion by the treating physician. Post-cholecystectomy diarrhea and BAD are known conditions, and after cholecystectomy, loss of the bile storage function may allow bile acids to flow into the intestine, thereby promoting water and electrolyte secretion in the colonic mucosa and causing diarrhea [1]. In addition, changes in the gut microbiota, altered bile acid metabolism, and increased intestinal motility mediated by bile acid-induced stimulation of intestinal serotonin have also been reported as possible mechanisms in post-cholecystectomy diarrhea [2].

In mid-April, the treating physician prescribed Questran powder 44.4%. The prescription was Questran powder 44.4% 18 g/day, twice daily after breakfast and dinner, for 14 days. The medication history available to the pharmacist included previous prescriptions of esomeprazole 20 mg once daily, acotiamide 100 mg three times daily, desloratadine 5 mg once daily, Biofermin tablets, domperidone 10 mg three times daily, fosfomycin 500 mg six tablets daily, and Bio-Three combination tablets. Based on the dispensing dates and prescribed durations, desloratadine was the only medication that may have overlapped with cholestyramine initiation. The other gastrointestinal medications, antibacterial agent, and probiotic or lactic acid bacterial preparations had been prescribed earlier in the clinical course and were considered prior treatments rather than confirmed concomitant medications at the time of cholestyramine initiation. Questran powder 44.4% is a powder formulation containing 4 g of cholestyramine per 9 g [4]. In Japan, the approved indications for Questran powder 44.4% include hypercholesterolemia, and its use for suspected BAD, as in this case, was off-label [4].

The pharmacist was not involved in the diagnosis or treatment selection by the treating physician. Instead, as support for appropriate use after the off-label prescription, the pharmacist reviewed drug information and overseas prescribing information. Cholestyramine binds bile acids in the intestine and promotes fecal excretion of bile acids [4,5]. The United Kingdom SmPC states that cholestyramine preparations are used for the relief of diarrhea associated with ileal resection, Crohn’s disease, vagotomy, and diabetic vagal neuropathy and that alternative therapy should be initiated if no response is observed within three days in diarrhea induced by bile acid malabsorption [5].

During the initial medication counseling, the pharmacist explained the administration method, dosing interval with concomitant medications, and the need for early confirmation of therapeutic response, taking into account that the drug was prescribed off-label in Japan. The United Kingdom SmPC states that Questran Light should not be taken in its dry powder form, that it should be mixed with water or another suitable liquid before administration, and that, when concomitant medications are used, they should be taken at least one hour before or four to six hours after Questran Light [5]. Based on this information, the pharmacist instructed the patient to take the medication as a suspension in water and to pay attention to the dosing interval with concomitant medications.

Before starting treatment, the patient-reported BSFS score was recorded as a range of 5-6, and stool frequency was five to six times per day. The BSFS is a 7-point scale for assessing stool form, and stool form is associated with changes in intestinal transit time. It is used in clinical practice and research as a simple indicator for evaluating changes in bowel function [6]. In this case, the pharmacist planned to evaluate the therapeutic response after treatment initiation using the BSFS and stool frequency.

Based on the United Kingdom SmPC, early assessment around day 3 after treatment initiation was initially planned [5]. However, because day 3 after treatment initiation fell on a weekend and it was difficult to contact the patient, the actual medication follow-up was conducted on day 5 after treatment initiation. At follow-up, the patient-reported BSFS score was recorded as a range of 4-5, and stool frequency had decreased to approximately two to three times per day. Thus, compared with the pretreatment status, improvements were observed in both stool form and frequency.

On the other hand, the patient reported difficulty taking Questran powder because of the texture of the suspension. Although the prescription instructed Questran powder 44.4% 18 g/day in two divided doses, the patient maintained the prescribed total daily amount of 18 g/day but took it in three divided administrations. The exact amount taken at each administration was not separately documented. Thus, the total daily amount was unchanged, but the dosing frequency differed from the prescribed twice-daily regimen. The pharmacist confirmed that symptom improvement had been observed, along with issues related to medication acceptability, and prepared a tracing report for the treating physician. The report included the improvements in BSFS score and stool frequency, actual medication use, and the possibility of considering a future change in dosage form or medication. No subsequent physician response, treatment modification, discontinuation, or documented reassessment after submission of the tracing report was available to the pharmacist.

The above course was organized as a practical process of pharmacist-led medication counseling following off-label prescribing, early follow-up, and the provision of information through a tracing report.

## Discussion

This case involved a patient with a history of cholecystectomy in whom suspected BAD was considered by the treating physician and for whom cholestyramine was prescribed for an indication that is off-label in Japan, after which a pharmacist became involved as support for appropriate use. The significance of this case does not lie in the pharmacist diagnosing BAD or verifying the efficacy of cholestyramine. Rather, it lies in the pharmacist reviewing domestic and overseas drug information for an off-label prescription related to a condition suspected by the treating physician, organizing the administration method and precautions, setting indicators for evaluating therapeutic response, assessing actual medication use, and providing this information to the physician through a tracing report.

The pharmacist’s role in this case can be organized into four main functions. First, the pharmacist reviewed domestic drug information on Questran powder 44.4% and the United Kingdom SmPC, and supplemented information on the pharmacological rationale, administration method, dosing interval with concomitant medications, and timing of therapeutic response assessment for a use that is off-label in Japan. Second, the pharmacist established a monitoring method to assess early therapeutic response after prescription using the BSFS and stool frequency. Third, through medication counseling and follow-up at the pharmacy, the pharmacist identified that the patient maintained the prescribed total daily amount of 18 g/day but was not taking the medication twice daily as prescribed and had instead divided it into three administrations. Fourth, the pharmacist shared changes in stool form and stool frequency, difficulty taking the medication, and actual medication use with the physician through a tracing report. These actions did not replace diagnosis or treatment selection. Rather, they represent a practical intervention in which the pharmacist, who had contact with the patient after prescription, used expertise in drug information and opportunities for continuous dialogue to support the safety and appropriateness of pharmacotherapy.

Post-cholecystectomy diarrhea and BAD are known conditions. After cholecystectomy, the storage function of the gallbladder is lost, making it easier for bile acids to flow from the small intestine into the colon, where they may promote water and electrolyte secretion in the colonic mucosa and cause diarrhea [1]. Changes in the gut microbiota and increased intestinal motility mediated by bile acid-induced stimulation of intestinal serotonin have also been reported as possible mechanisms [2]. However, in this case, approximately 18 years had passed since cholecystectomy, and suspected BAD was not definitively confirmed by specific diagnostic testing. In the information available to the pharmacist from routine community pharmacy practice, no results of relevant laboratory or diagnostic investigations for persistent diarrhea were available, including stool culture, blood tests, abdominal imaging, colonoscopy, SeHCAT testing, serum 7α-hydroxy-4-cholesten-3-one, fibroblast growth factor 19, or 48-hour fecal bile acid measurement. On the other hand, considering the history of cholecystectomy, persistence of symptoms after ingestion of high-fat foods, and a clinical course that was not typical of infectious gastroenteritis, it is reasonable to position this as a case in which the treating physician included BAD in the differential diagnosis. Although several diagnostic tests are available for BAD, access to and feasibility of these tests may be limited in clinical practice, and therapeutic trials with bile acid sequestrants may be used [1].

In Japan, the approved indications for Questran powder 44.4% include hypercholesterolemia, and its use for suspected BAD, as in this case, is off-label [4]. In contrast, the United Kingdom SmPC states that Questran Light is used for the relief of diarrhea associated with ileal resection, Crohn’s disease, vagotomy, and diabetic vagal neuropathy, and that alternative therapy should be initiated if no response is observed within three days in diarrhea induced by bile acid malabsorption [5]. This information served as the basis for the pharmacist to plan early follow-up in this case and to assess therapeutic response within a short period rather than allowing treatment to continue without structured evaluation.

In this case, based on the statement in the United Kingdom SmPC regarding early response assessment, the pharmacist planned medication follow-up soon after treatment initiation [5]. In practice, because day 3 after treatment initiation fell on a weekend and it was difficult to contact the patient, follow-up was conducted on day 5 after treatment initiation. Before treatment, the BSFS score was 5-6, and stool frequency was five to six times per day. At follow-up, the BSFS score had improved to 4-5, and stool frequency had decreased to approximately two to three times per day. This improvement was an observation in a single case and does not generalize the efficacy of cholestyramine. However, the case demonstrates the practical significance of pharmacist-led post-prescription monitoring in that the pharmacist assessed symptom changes early after prescription and provided clinically relevant information to the physician through a tracing report.

The BSFS and stool frequency were used to evaluate therapeutic response. The BSFS is a 7-point scale for assessing stool form, and stool form is associated with changes in intestinal transit time. It is used in clinical practice and research as a simple indicator for monitoring changes in bowel function [6]. In this case, the BSFS functioned as an auxiliary indicator to convert the patient’s subjective statement that “diarrhea improved” into standardized information that could be shared with the physician. Only patient BSFS scores were reported in this manuscript; the BSFS visual scale, chart, and other copyrighted scale content were not reproduced. However, the BSFS is based on patient self-report and does not directly measure fecal bile acid levels or intestinal transit time. Therefore, in this case, the BSFS was positioned not as a diagnostic indicator but as an auxiliary tool for longitudinal assessment of therapeutic response and information sharing.

Early follow-up identified not only therapeutic response but also problems with medication use. The SmPC for Questran Light states that the drug should not be taken in its dry powder form, that it should be mixed with water or another suitable liquid before administration, and that concomitant medications should be taken at least one hour before or four to six hours after Questran Light [5]. Based on this information, the pharmacist explained how to take the medication as a suspension and how to separate its administration from other medications.

However, the patient reported difficulty taking the medication because of the texture of the suspension. Although the prescription instructed Questran powder 44.4% 18 g/day in two divided doses, he maintained the prescribed total daily amount of 18 g/day but took it in three divided administrations. This information represents an aspect of actual medication use that would have been difficult to identify without medication follow-up, and it demonstrates the significance of pharmacist involvement through ongoing opportunities for dialogue at the pharmacy counter or by telephone. In addition, because the actual administration schedule differed from the prescription, the observed symptom improvement in this case cannot be evaluated as a response to the prescribed twice-daily regimen. The pharmacist shared this actual medication use with the physician through a tracing report, together with the changes in stool form and stool frequency.

In Japan, a case report has described successful treatment of diabetic diarrhea with cholestyramine, suggesting that symptom improvement may have been attributable to cholestyramine binding bile acids in the intestine and suppressing their laxative effect [7]. However, that report was a physician-authored case report of diabetic diarrhea, and the underlying condition differs from the present case, which involved a history of cholecystectomy. It also does not provide a direct comparison with the practical process in this case, namely pharmacist-led information supplementation after off-label prescribing, medication follow-up, and information provision to the physician through a tracing report. Therefore, although that report is useful as a domestic example of cholestyramine use for diarrheal symptoms, it has limitations as a comparator for the pharmacist practice that is the main focus of this case.

In Japan, clinical guidelines for chronic diarrhea have been developed, and efforts have been made to systematically organize the diagnosis and treatment of chronic diarrhea [3]. While definitive diagnosis and treatment decisions are made by physicians, pharmacists can contribute to support for appropriate use after prescription, confirmation of medication use, identification of adverse events and medication-related problems, and sharing of therapeutic response. This case is a practical example in which the pharmacist did not perform diagnostic activity but instead collected and evaluated domestic and overseas drug information for an off-label prescription, conducted early follow-up using standardized symptom indicators, and provided information to the physician through a tracing report. Such a process may support structured assessment after off-label prescribing by organizing observed symptom changes and medication-related issues, rather than allowing off-label prescriptions to continue without follow-up. However, no subsequent physician response, treatment modification, discontinuation, or documented reassessment after submission of the tracing report was available to the pharmacist. Therefore, this case cannot determine the direct impact of the tracing report on the physician’s decision-making or on the long-term outcome.

Several limitations should be considered when interpreting this case. First, BAD was clinically suspected, but no definitive diagnosis was made using specific diagnostic tests. Second, a long period had passed since cholecystectomy, and a causal relationship between the current diarrheal symptoms and cholecystectomy cannot be clearly demonstrated. Third, before starting cholestyramine, the patient had received probiotics, antibacterial therapy, and lactic acid bacterial preparations, and the effects of spontaneous improvement or regression to the mean cannot be completely excluded. Therefore, the improvement observed after treatment initiation cannot be attributed solely to the pharmacological effect of cholestyramine. Fourth, although the patient maintained the prescribed total daily amount of 18 g/day, he did not take the medication twice daily as prescribed but divided it into three administrations, making it difficult to evaluate the effect of the prescribed twice-daily administration schedule based on this case. Fifth, both the BSFS and stool frequency depended on patient self-report, and measurement error or reporting bias may have been present. Furthermore, because this was a single case, the efficacy of cholestyramine and the usefulness of pharmacist-led early follow-up cannot be generalized.

In summary, the significance of this case does not lie in showing that cholestyramine itself was effective in a patient with suspected BAD. Rather, it lies in presenting a practical process in which, during off-label pharmacotherapy, the pharmacist supplemented drug information including overseas SmPC information, assessed therapeutic response early using the BSFS and stool frequency, identified the difference between the prescribed regimen and actual medication use, and shared this information with the physician through a tracing report. This approach may serve as one method in community pharmacy practice for supporting the safety and appropriateness of off-label prescribing through early monitoring, identification of medication-use problems, and communication with the treating physician.

## Conclusions

This case involved a patient with a history of cholecystectomy in whom suspected BAD was included in the differential diagnosis, and to whom cholestyramine, which is off-label in Japan for this purpose, was prescribed. After the prescription, the pharmacist became involved as support for appropriate use. The pharmacist was not involved in confirming the diagnosis of BAD or in selecting cholestyramine. After the off-label prescription was issued, the pharmacist reviewed domestic drug information and overseas prescribing information, organized the pharmacological rationale, administration method, dosing interval with concomitant medications, and the need for early assessment of symptom changes, and provided medication counseling.

In addition, by using the BSFS and stool frequency, the pharmacist organized the patient’s symptom changes in a form that could be shared with the physician, and also identified aspects of actual medication use, including difficulty taking the suspension and the difference between the prescribed twice-daily regimen and the patient’s actual method of administration. This information was provided to the physician through a tracing report. However, the improvement observed in this case was based on a single case, and the effects of prior treatments and the deviation from the prescribed twice-daily administration schedule could not be excluded. In addition, the direct impact on the physician’s decision regarding treatment continuation was not evaluated. Therefore, interpretation of this case has limitations. This case shows a practical example in which, in off-label prescribing, the pharmacist did not replace diagnosis or treatment decision-making but supported the appropriate use of pharmacotherapy through early post-prescription monitoring, identification of medication-use problems, assessment of actual medication use, and communication with the treating physician through a tracing report.
